# Promoting Persistent Superionic Conductivity in Sodium Monocarba-*closo*-dodecaborate NaCB_11_H_12_ via Confinement within Nanoporous Silica

**DOI:** 10.1021/acs.jpcc.1c03589

**Published:** 2021-07-26

**Authors:** Mikael S. Andersson, Vitalie Stavila, Alexander V. Skripov, Mirjana Dimitrievska, Malgorzata T. Psurek, Juscelino B. Leão, Olga A. Babanova, Roman V. Skoryunov, Alexei V. Soloninin, Maths Karlsson, Terrence J. Udovic

**Affiliations:** † Department of Chemistry and Chemical Engineering, Chalmers University of Technology, SE-412 96 Göteborg, Sweden; ‡ Department of Chemistry, Ångström Laboratory, Uppsala University, Box 538, 75121 Uppsala, Sweden; § NIST Center for Neutron Research, National Institute of Standards and Technology, Gaithersburg, Maryland 20899-6102, United States; ∥ Energy Nanomaterials, Sandia National Laboratories, Livermore, California 94551, United States; ⊥ Institute of Metal Physics, Ural Branch of the Russian Academy of Sciences, Ekaterinburg 620108, Russia; # National Renewable Energy Laboratory, Golden, Colorado 80401, United States; ∇ Laboratory of Semiconductor Materials, Institute of Materials, Ecole Polytechnique Fédérale de Lausanne, 1015 Lausanne, Switzerland; ○ Department of Chemistry, University of Maryland, College Park, Maryland 20742, United States; ◆ Department of Materials Science and Engineering, University of Maryland, College Park, Maryland 20742, United States

## Abstract

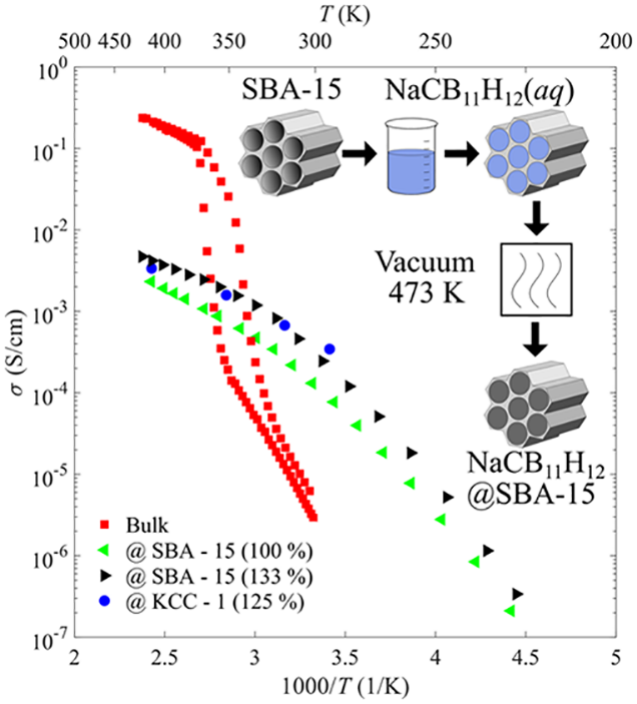

Superionic phases of bulk anhydrous salts based on large cluster-like polyhedral (carba)borate anions are generally stable only well above room temperature, rendering them unsuitable as solid-state electrolytes in energy-storage devices that typically operate at close to room temperature. To unlock their technological potential, strategies are needed to stabilize these superionic properties down to subambient temperatures. One such strategy involves altering the bulk properties by confinement within nanoporous insulators. In the current study, the unique structural and ion dynamical properties of an exemplary salt, NaCB_11_H_12_, nanodispersed within porous, high-surface-area silica via salt-solution infiltration were studied by differential scanning calorimetry, X-ray powder diffraction, neutron vibrational spectroscopy, nuclear magnetic resonance, quasielastic neutron scattering, and impedance spectroscopy. Combined results hint at the formation of a nanoconfined phase that is reminiscent of the high-temperature superionic phase of bulk NaCB_11_H_12_, with dynamically disordered CB_11_H_12_^–^ anions exhibiting liquid-like reorientational mobilities. However, in contrast to this high-temperature bulk phase, the nanoconfined NaCB_11_H_12_ phase with rotationally fluid anions persists down to cryogenic temperatures. Moreover, the high anion mobilities promoted fast-cation diffusion, yielding Na^+^ superionic conductivities of ∼0.3 mS/cm at room temperature, with higher values likely attainable via future optimization. It is expected that this successful strategy for conductivity enhancement could be applied as well to other related polyhedral (carba)borate-based salts. Thus, these results present a new route to effectively utilize these types of superionic salts as solid-state electrolytes in future battery applications.

## Introduction

Since the first report of superionic conductivity observed in Na_2_B_12_H_12_ above its order–disorder phase transition,^1^ a rich variety of similarly superionic Li^+^, Na^+^, and Ag^+^ solid-state electrolytes have been identified based on the anhydrous salts of the large cluster-like polyhedral (carba)borate anions.^2−8^ The critical factor promoting the superionic state is the formation of an entropy-driven disordered phase of rapidly reorienting anions among translationally fluidic interstitial cations, which normally occurs somewhere above room temperature, below which the ordered salt is poorly conductive. For practical battery applications, strategies are required to stabilize the superionic disordered phases at room temperature and below. Ball-milling these salts to create nanosized domains^9^ and combining salts with different polyhedral anions^9−13^ are two methods that have proven to be successful for stabilizing the desired disordered phases down to subambient temperatures. Here, we exemplify a third viable method, that is, the nanodispersion of polyhedral (carba)borate salts within porous, high-surface-area silica (SiO_2_) via salt-solution infiltration with subsequent vacuum desolvation. Somewhat analogous melt-infiltration of pure and anion-substituted LiBH_4_ in nanostructured SiO_2_ and Al_2_O_3_ has been shown to yield highly Li^+^-conductive nanocomposites.^14−16^

In addition to these LiBH_4_-related examples, there have been at least two reported attempts to create similarly nanoconfined polyhedral (carba)borate salts, in particular, involving Li_2_B_12_H_12_. In one case, further reaction of SBA-15 SiO_2_-nanoconfined LiBH_4_ with an H_2_/B_2_H_6_ gas mixture at 433 K was found to produce Li_2_B_12_H_12_ (with ∼6% Li_2_B_10_H_10_) within the (5.9 nm average diameter) cylindrical nanopores.^17^ Moreover, despite the apparently successful formation of a nanoconfined Li_2_B_12_H_12_ reaction product, the resulting nanocomposite material displayed similarly low, ambient-temperature conductivities as bulk Li_2_B_12_H_12_, reflecting the observed dominance within the nanopores of the ordered α-Li_2_B_12_H_12_ phase that normally forms in the bulk at these temperatures. Despite this main ordered phase, the structural measurements could not rule out that a small fraction of the nanoconfined Li_2_B_12_H_12_ was in its highly conductive, disordered β-phase. ^7^Li nuclear magnetic resonance (NMR) also indicated the coexistence of temperature-independent fractions of more and less mobile Li^+^ cations, although it is not clear to what extent the more mobile fraction is related to the presence of some β-phase and/or is associated with a perturbed Li_2_B_12_H_12_ layer adjacent to the nanopore wall. In another subsequent study, it was found that solid crystalline adducts of Li_2_B_12_H_12_ with either acetonitrile (ACN) or tetrahydrofuran (THF) melt below 423 K during partial desolvation, and this allowed for the melt infiltration of Li_2_B_12_H_12_·*x*ACN and Li_2_B_12_H_12_·*x*THF into SBA-15 nanoporous SiO_2_.^18^ Further evacuation above ∼493 K was suggested by X-ray diffraction techniques to yield nanoconfined, solvent-free, solidified Li_2_B_12_H_12_, presumably with an α-phase signature. However, the resulting Li^+^ conductivity behavior of a Li_2_B_12_H_12_·*x*THF-derived nanocomposite was found to be unremarkable and akin to that of the previously reported^17^ impure Li_2_B_12_H_12_@SiO_2_ nanocomposites.

Unfortunately, these latter results do not firmly resolve whether nanoconfinement can help stabilize favorable nonbulk-like properties with enhanced Li^+^ or other ion conductivities for any of the general class of polyhedral (carba)borate salts, as it has for LiBH_4_ and its derivatives. We address this in the current study, where we turn our attention away from Li-based polyhedral (carba)borate salts and toward Na-based ones. In particular, sodium monocarba-*closo-*dodecaborate, NaCB_11_H_12_, was chosen in the present case based on its superior conductivity in the disordered state^3^ and its propensity to form highly concentrated aqueous solutions, which facilitates higher salt loadings within the pores during the salt-solution infiltration step.

## Experimental Details

**Figure 1:**
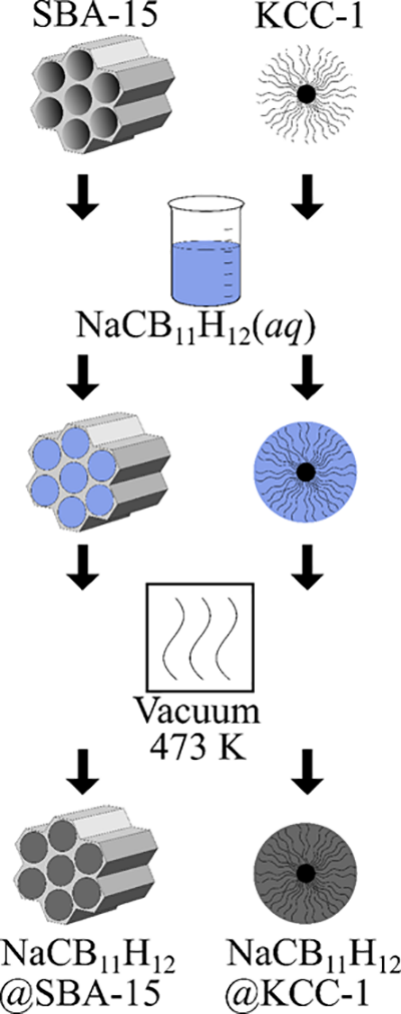
Schematic representation of the two nanostructures and the aqueous salt infiltration procedure.

NaCB_11_H_12_ was obtained from Katchem.^19^ Two morphologically distinct silica (SiO_2_) scaffolds, shown schematically in Figure 1, were tested: (1) SBA-15 (Sigma Aldrich) hexagonal honeycomb arrays of nominally 8 nm diameter tubular nanopores (with a reported pore volume of ∼0.8–1.0 cm^3^ g^–1^ and a surface area of 450–550 m^2^ g^–1^) and (2) KCC-1 (Strem) high-surface-area (700 m^2^ g^–1^) spherical fibrous SiO_2_ nanoparticles with interconnected nanoporous volume within the fiber network. Silica powders from the sealed vendor bottles were typically first evacuated at 473 K for 16 h to remove trace H_2_O, although the lack of this step before infiltration was found not to affect the ultimate results. These powders were wetted at room temperature with chosen amounts of saturated aqueous NaCB_11_H_12_ solution (approximately 5.5 H_2_O molecules per NaCB_11_H_12_ formula unit) using a micropipette. After stirring the slurry mixtures to ensure uniform infiltration via capillary action, H_2_O was removed by vacuum evacuation at 473 K for 16 h. Additional anhydrous NaCB_11_H_12_ for comparative bulk measurements was also generated using this drying procedure. After drying, all materials were handled in a He-filled glovebox.

Powder X-ray diffraction (PXRD) patterns were measured for each sample sealed in 1 mm diameter quartz capillaries at room temperature using a Rigaku Ultima III X-ray diffractometer with a Cu-Kα source (λ = 1.5418 Å). Differential scanning calorimetry (DSC) measurements with thermogravimetric analysis (TGA) were performed with a Netzsch (STA 449 *F1 Jupiter*) TGA–DSC under He flow using Al sample pans.

Low-field proton NMR measurements were performed on a pulse spectrometer described earlier^20^ at the frequencies ω/2π = 14 and 28 MHz. Typical values of the π/2 pulse length were 2–3 μs. High-field ^23^Na NMR measurements were performed with a Bruker AVANCE III 500 spectrometer at the frequency ω/2π = 132.3 MHz. Nuclear spin–lattice relaxation rates were measured using the saturation–recovery method. NMR spectra were recorded by Fourier-transforming the solid echo signals (pulse sequence π/2*_x_* – *t* – π/2*_y_*). NMR samples were flame-sealed in quartz tubes under vacuum.

Neutron scattering measurements were performed at the National Institute of Standards and Technology Center for Neutron Research using thin flat-plate-shaped samples to minimize neutron beam attenuation from the highly neutron-absorbing ^10^B that comprises 20% of natural boron. Neutron vibrational spectroscopy (NVS) measurements were performed with the Filter-Analyzer Neutron Spectrometer (FANS)^21^ using a Cu(220) monochromator with pre- and postcollimations of 20′ of arc, yielding a full-width-at-half-maximum (FWHM) energy resolution of about 3% of the neutron energy transfer. Neutron-elastic-scattering fixed-window scans (FWSs) were performed between 120 and 420 K in heating and cooling regimes at ±0.25 K min^–1^ with the High-Flux Backscattering Spectrometer (HFBS),^22^ which provides an energy resolution of 0.8 μeV FWHM using 6.27 Å wavelength incident neutrons. Quasielastic neutron scattering (QENS) measurements were performed with the DisKChopper Spectrometer (DCS),^23^ utilizing incident neutron wavelengths of 8 Å (1.278 meV) and 10 Å (0.818 meV), with resolutions of 29.8 and 17 μeV FWHM, respectively. QENS measurements below room temperature were performed using HFBS. The instrumental resolution functions on both instruments were obtained from the purely elastic QENS measurements at 4 K. Neutron data analyses were performed using the DAVE software package.^24^

Alternating current (AC) impedance measurements were performed under a He atmosphere. The electrolyte powders were pressed into free-standing pellets with a diameter of 5 mm and thicknesses of about 2 mm. The pellets were formed under 250 MPa uniaxial pressure using a miniature button compression load cell from OMEGA and loaded into an MTI stainless steel cell with Au electrodes on both sides of the pellet. Once loaded, the MTI cell applies a pressure of approximately 2.7 MPA on the pellet. The measurements were performed at temperatures from 225 to 420 K using a closed-cycle He refrigerator. The sample temperature was measured using a calibrated thermocouple. The AC impedance spectra were collected from 1 Hz to 1 MHz using a VersaSTAT 4 potentiostat.

All structural depictions were made using the VESTA (visualization for electronic and structural analysis) software.^25^ For all figures, standard uncertainties are commensurate with the observed scattering in the data, if not explicitly designated by vertical error bars.

## Results and Discussion

### Pore Loading and Structural Characterization

DSC was used for establishing the maximum amount of NaCB_11_H_12_ that could be incorporated into the nanoporous silica structures via solution-loading because of the different calorimetric behaviors found for bulk and nanoconfined NaCB_11_H_12_. While bulk NaCB_11_H_12_ exhibits a reversible DSC peak (near 380 and 354 K upon heating and cooling, respectively) that reflects the enthalpic change occurring during its transition back and forth between its poorly conductive, ordered orthorhombic and superionic, disordered phases,^3^ nanoconfined NaCB_11_H_12_ was found to manifest no such enthalpic feature over the entire probed temperature range (100 to 473 K).

**Figure 2:**
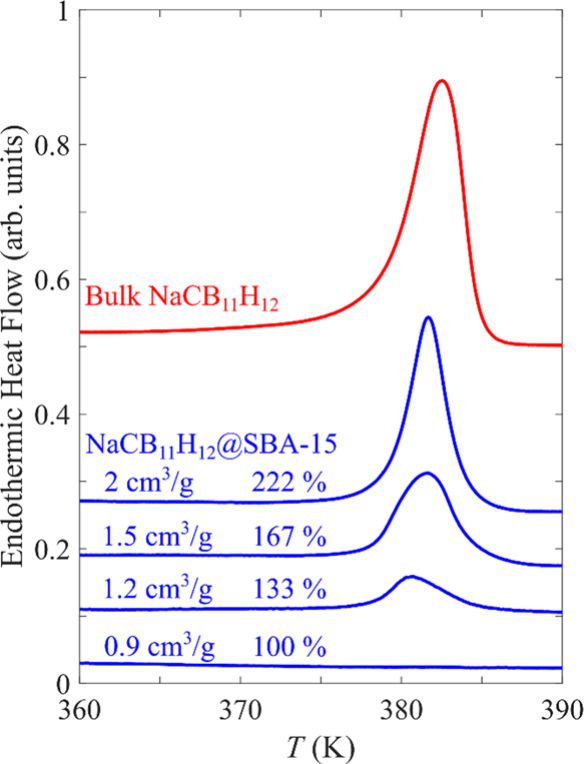
Exemplary DSC scans in heating (5 K min^–1^) of dried NaCB_11_H_12_@SBA-15 nanocomposite samples derived from different saturated NaCB_11_H_12_ solution loadings (cm^3^g^–1^ and corresponding percentage of available pore volume filled) of SBA-15, compared with that for bulk NaCB_11_H_12_. All nanocomposite scans are normalized with respect to the same mass of SBA-15.

Samples loaded with different solution volumes (cm^3^) per gram of both SBA-15 and KCC-1 silica were prepared. DSC temperature-cycling measurements of the resulting dried nanocomposites lacked any observable enthalpic features for samples with solution loadings at or below ∼0.9 and ∼1.2 cm^3^ g^–1^ for SBA-15 and KCC-1, respectively. In contrast, a bulk-like feature appeared for the higher-loading samples, with intensity increasing in a roughly linear fashion in proportion to the excess amount of solution added above the aforementioned values. An exemplary series of DSC scans for NaCB_11_H_12_@SBA-15 and NaCB_11_H_12_@KCC-1 in heating compared to that for bulk NaCB_11_H_12_ is shown in Figures 2 and S1, respectively. (Additional representative DSC cycling scans in both heating and cooling are shown in Figure S2.) These results suggest that all NaCB_11_H_12_ resides in the nanopores (exhibiting atypical NaCB_11_H_12_ calorimetric properties) after drying nanocomposites with solution loadings below these values. The cutoff at 0.9 cm^3^ g^–1^ for SBA-15 is in line with its reported accessible pore volume; the higher 1.2 cm^3^ g^–1^ cutoff for KCC-1 is also reasonable, considering its different morphology and associated 30–40% higher specific surface area compared to SBA-15, which is in agreement with the accessible KCC-1 pore volume reported by others.^26,27^ Henceforth, in subsequent discussion, we define 100% solution loading as 0.9 cm^3^ g^–1^ for SBA-15 and 1.2 cm^3^ g^–1^ for KCC-1.

**Figure 3:**
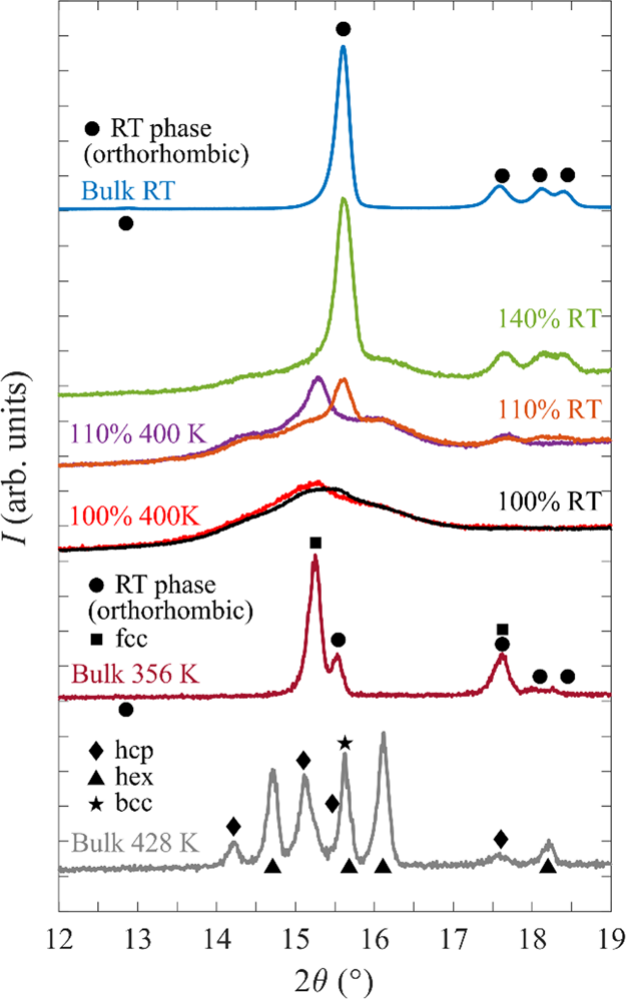
PXRD patterns for dried NaCB_11_H_12_@SBA-15 nanocomposites for different solution loadings and measurement temperatures (100% loading, room temperature (RT): black; 100%, 400 K: red; 110%, RT: brown; 110%, 400 K: purple; and 140% RT: green) compared with bulk NaCB_11_H_12_ patterns (RT: blue, 356 K: maroon; and 428 K: gray) from a previous study.^3^ The Bragg positions of the bulk ordered orthorhombic phase are marked with black circles, whereas those for the various known disordered fcc, hcp, hexagonal, and bcc polymorphs are marked by black squares, diamonds, triangles, and stars, respectively.

Figure 3 shows the diffraction patterns for the ordered orthorhombic and disordered cubic and hexagonal phases of bulk NaCB_11_H_12_ compared with those for dried NaCB_11_H_12_@SiO_2_ nanocomposites corresponding to various NaCB_11_H_12_ solution loadings in SBA-15. At 100% solution loading, the room-temperature pattern for the resulting nanosequestered NaCB_11_H_12_ displays one predominant, considerably broadened Bragg feature centered near 15.3°, which is in line with the main Bragg peak of the high-temperature face-centered-cubic (fcc) phase of bulk NaCB_11_H_12_ that was found to dominate at 356 K (with some remnant ordered orthorhombic phase).^3^ Moreover, there are indications of minor broad sidebands near 14.3 and 16.1°, which is consistent with the Bragg peak positions of two other higher-temperature polymorphs of bulk NaCB_11_H_12_ (with hexagonal symmetries) that were found to dominate at 428 K.^3^ Even the nonsymmetric shape surrounding the peak maximum may be an indication of an additional minor feature at 15.6°, which would match the fourth higher-temperature polymorph of bulk NaCB_11_H_12_ (with body-centered-cubic symmetry) that was also observed at 428 K.^3^ We note that the broadening of the peaks implies that the crystallites of NaCB_11_H_12_ are indeed nanosized, as expected.

For 110 and 140% loadings, on the top of the broadened Bragg feature, it is clear that additional sharp Bragg peaks representing the ordered orthorhombic phase of excess bulk NaCB_11_H_12_ outside the pores are present at room temperature and become more intense with increased loading. Moreover, as seen by the 400 K pattern for 110% loading, the sharp orthorhombic Bragg peaks for the excess bulk NaCB_11_H_12_ are replaced by the two expected peaks representing the high-temperature fcc phase, while little change is evident for the underlying broad Bragg feature because of the nanoconfined NaCB_11_H_12_. Analogous PXRD patterns for the KCC-1 infiltrated samples are exemplified in Figure S3. The results are similar, although the fraction of the fcc polymorph in the pores appears to be lower than that in SBA-15 at the expense of the other known disordered polymorphs for NaCB_11_H_12_ highlighted in Figure 3. This is possibly due to a crystallite-size effect because the average pore size (as well as the pore size distribution) in KCC-1 has been shown to be larger than the distinct tubular 8 nm pores of SBA-15. Indeed, in addition to a minor fraction of smaller nanopores (<4–5 nm), the majority of the KCC-1 pore volume typically exists as mesopores ranging from roughly 5 nm to 25–30 nm.^26^ This is consistent with the fact that the disordered polymorph PXRD features for NaCB_11_H_12_@KCC-1 appear to be slightly sharper than those for NaCB_11_H_12_@SBA-15, reflecting the larger average crystallite size for the former.

Neutron vibrational spectra at 4 K for bulk NaCB_11_H_12_ and both NaCB_11_H_12_@SBA-15 and NaCB_11_H_12_@KCC-1 (100% loadings) shown in Figure S4 indicate similar optical phonon densities of states characteristic of the various CB_11_H_12_^–^ anion deformation modes.^3^ In short, the combined DSC, PXRD, and NVS results indicate that NaCB_11_H_12_ remains nanoconfined and undecomposed after drying the NaCB_11_H_12_-solution-infiltrated SiO_2_. Moreover, although not conclusive, PXRD suggests the stabilization of room-temperature nanostructures similar to that of the disordered structures seen only at higher temperatures for bulk NaCB_11_H_12_. Because PXRD is only sensitive to ordered structures, we cannot rule out the existence of additional “invisible” amounts of amorphous-like NaCB_11_H_12_ within the pores, although the relative intensities of the Bragg features for bulk NaCB_11_H_12_ and disordered polymorphs in overloaded NaCB_11_H_12_@SiO_2_ samples suggest that the amount of any such amorphous material is very minor at best. Moreover, the preferred formation of normally higher-temperature, bulk-like polymorphs for nanoconfined NaCB_11_H_12_ at room temperature and below is not unexpected, as evidenced by the similar structural transformation of coarsely crystalline NaCB_11_H_12_ upon entering the nanocrystalline regime via ball-milling.^9^ The lack of an obvious DSC enthalpic feature down to 100 K further suggests that these nanostructures remain stable down to cryogenic temperatures. Finally, at higher solution loadings exceeding the accessible SiO_2_ pore volume, both DSC and PXRD indicate that excess NaCB_11_H_12_ crystallizes outside the pore as bulk phase.

### Anion and Cation Dynamics

**Figure 4:**
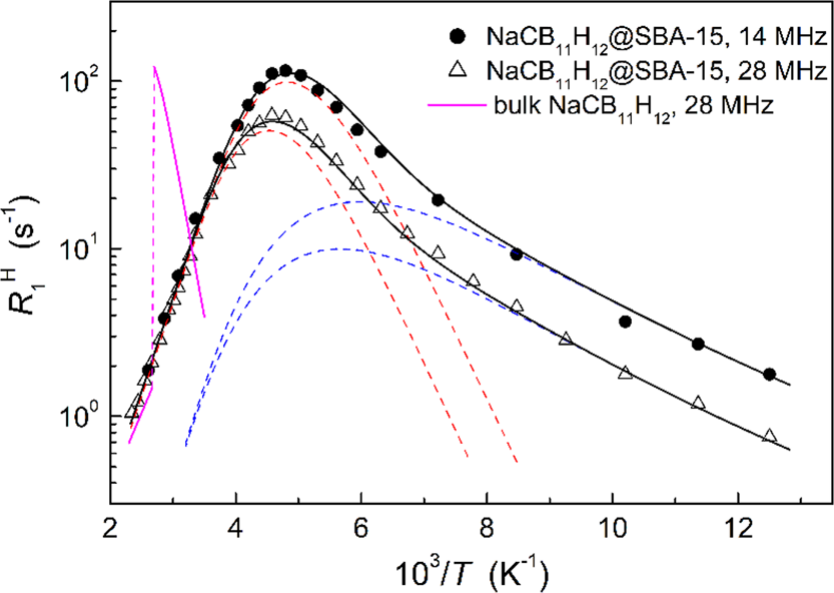
Proton spin–lattice relaxation rates measured at 14 and 28 MHz for NaCB_11_H_12_@SBA-15 (100% loading) as functions of the inverse temperature. The black solid lines show the results of the simultaneous fit of a two-peak model to the data in the range 80–426 K. The dashed lines show the contributions of the faster (blue) and slower (red) jump processes to the fit. For comparison, the magenta lines show the behavior of the proton spin–lattice relaxation rate at 28 MHz in bulk NaCB_11_H_12_ (from ref (31)).

The dynamical behaviors of the CB_11_H_12_^–^ anions and Na^+^ cations of the (100% loading) NaCB_11_H_12_@SBA-15 SiO_2_ nanocomposite were probed by ^1^H and ^23^Na NMR measurements to understand better the effects of nanoconfinement. Figure 4 shows the proton spin–lattice relaxation rates, *R*_1_^H^, at ω/2π = 14 and 28 MHz as functions of the inverse temperature. As in other polyhedral hydro(carba)borate salts,^28^ the behavior of *R*_1_^H^ in NaCB_11_H_12_@SBA-15 is governed by reorientations of the complex anions via the mechanism of motionally modulated dipole–dipole interactions between nuclear spins.^29^ For this mechanism, *R*_1_^H^(*T*) is expected to pass through a maximum at the temperature where the H jump rate τ ^–1^ becomes nearly equal to the resonance frequency ω. For NaCB_11_H_12_@SBA-15, this occurs at 210 K (∼10^8^ s^–1^). It should be noted that the progression of the relaxation rate curve at lower temperatures shown in Figure 4 suggests the coexistence of an additional faster reorientational jump process, not unexpected based on the molecular symmetry of the dipolar CB_11_H_12_^–^ anion. Indeed, a similar relaxation rate behavior reflecting coexisting jump processes was also observed for superionic Na_2_(CB_9_H_10_)(CB_11_H_12_), with the maximum in *R*_1_^H^ occurring near 220 K for the orientationally disordered CB_9_H_10_^–^ and CB_11_H_12_^–^ anions in the hexagonal structure.^10,30^ This behavior for nanoconfined NaCB_11_H_12_ is in sharp contrast to that for bulk NaCB_11_H_12_, where *R*_1_^H^(*T*) does not reach a maximum even upon heating to 376 K.^31^ Instead, the proton relaxation rate of bulk NaCB_11_H_12_ exhibits an abrupt two-orders-of-magnitude drop at 376 K, which relates to the order–disorder phase transition, leading to the strong acceleration of the reorientational motion.^31^ The lack of such a rapid change in the jump rate points to the stabilization of bulk-like disordered phases down to cryogenic temperatures, which is in agreement with the DSC and PXRD results. In particular, the order–disorder phase transition normally occurring for bulk NaCB_11_H_12_ appears to be suppressed so that high reorientational mobility is retained down to low temperatures.

**Figure 5:**
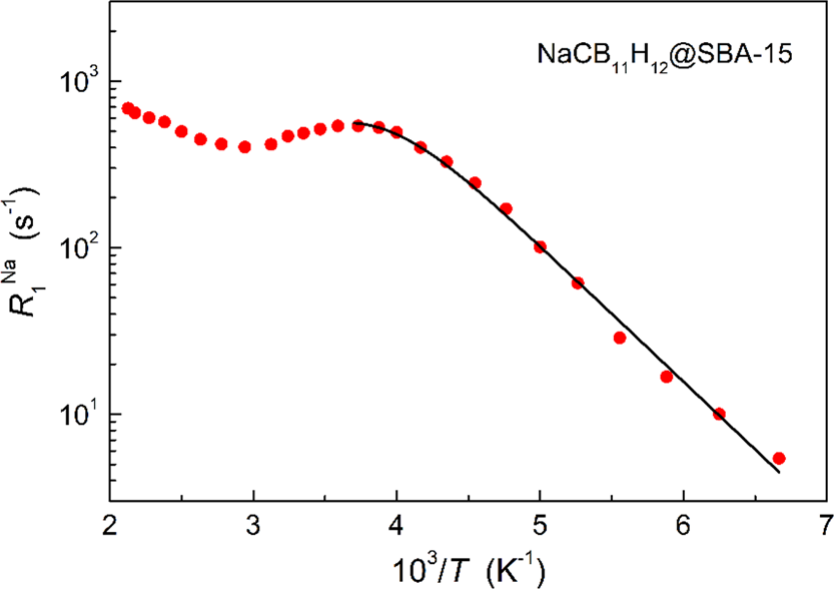
^23^Na spin–lattice relaxation rate measured at 132 MHz for NaCB_11_H_12_@SBA-15 (100% loading) as a function of the inverse temperature. The solid line shows the model fit (eq. S2 in the Supporting Information) to the data in the range 150–270 K.

Figure 5 shows the measured ^23^Na spin–lattice relaxation rate *R*_1_^Na^ as a function of the inverse temperature. In the temperature range 150–320 K, *R*_1_^Na^ exhibits a behavior typical of the motionally induced spin–lattice relaxation with the characteristic peak near 270 K, indicating that the diffusive Na^+^ jump rate τ_d_^–1^ reaches approximately ω ∼8 × 10^8^ s^–1^ at this temperature. An activation energy of 162(4) meV for the Na^+^ diffusive motion, *E*_a_^d^, was extracted by fitting the *R*_1_^Na^(*T*) data in the temperature range 150–270 K; see Figure 5. Further details are given in the Supporting Information. In addition to the *R*_1_^Na^(*T*) peak near 270 K, there is an increase in *R*_1_^Na^(*T*) above 330 K, suggesting that there may be a second high-temperature relaxation rate peak, and thus, a second (slower) Na^+^ jump process may coexist with the faster Na^+^ jump process responsible for the *R*_1_^*Na*^(*T*) peak near 270 K.

**Figure 6:**
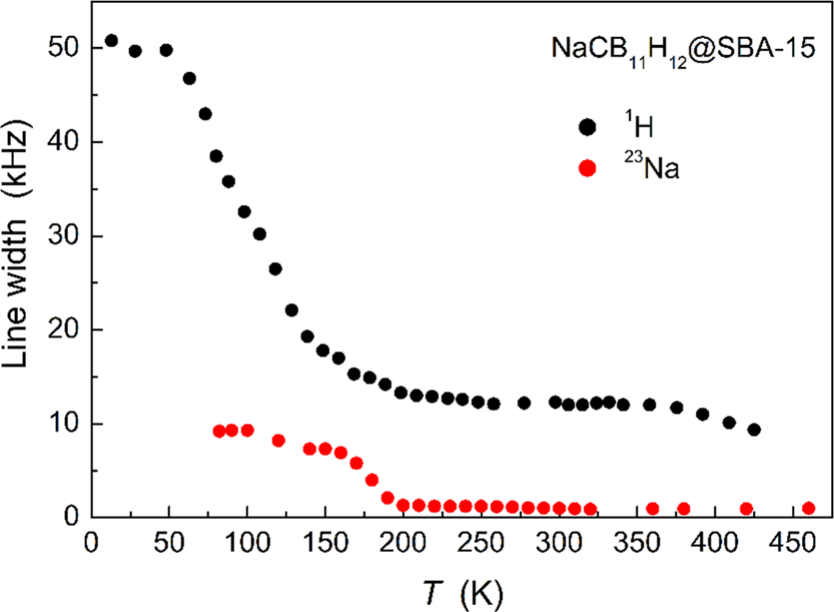
Temperature dependences of the ^1^H and ^23^Na NMR line widths (FWHM) measured for NaCB_11_H_12_@SBA-15 (100% loading) at 28 MHz (^1^H) and 132 MHz (^23^Na).

The evolution of the ^1^H and ^23^Na NMR line widths (Δ_H_ and Δ_Na_) as a function of temperature for NaCB_11_H_12_@SBA-15 is shown in Figure 6. At low temperatures, Δ_H_ is determined by dipole–dipole interactions in the “rigid” lattice and is similar to the “rigid–lattice” Δ_H_ values (∼50 kHz) of other polyhedral hydro(carba)borate salts.^20,31^ With increasing temperature, Δ_H_ starts to decrease because the dipole–dipole interactions between nuclear spins are partially averaged by the reorientational motion of the anions. A considerable decrease in Δ_H_ is expected when τ^–1^ becomes comparable to the “rigid–lattice” linewidth,^29^ which can be seen to occur near 100 K for the NaCB_11_H_12_@SBA-15 nanocomposite. This indicates that the reorientational jump rate reaches ∼3 × 10^5^ s^–1^ at this temperature. A typical feature of complex hydrides with reorienting anions is that Δ_H_ reaches a significant nonzero plateau value at higher temperatures.^32^ This indicates that the motion is a *localized* H motion (e.g., anion reorientations) because the dipole–dipole interactions of ^1^H spins are not fully averaged by such motions, which leads to an offset.

For Δ_Na_, a strong narrowing near 180 K can be observed, indicating the onset of fast diffusive motion of Na^+^ cations with jump rates exceeding ∼10^4^ s^–1^. While the Δ_H_ high-temperature plateau value exhibits a significant offset, the Δ_Na_ high-temperature plateau value is close to zero (∼ 0.95 kHz). This indicates that the Na^+^ cations participate in the *long-range* diffusion, which leads to an averaging out of both the dipole–dipole and quadrupole interactions of the ^23^Na nuclei.

**Figure 7:**
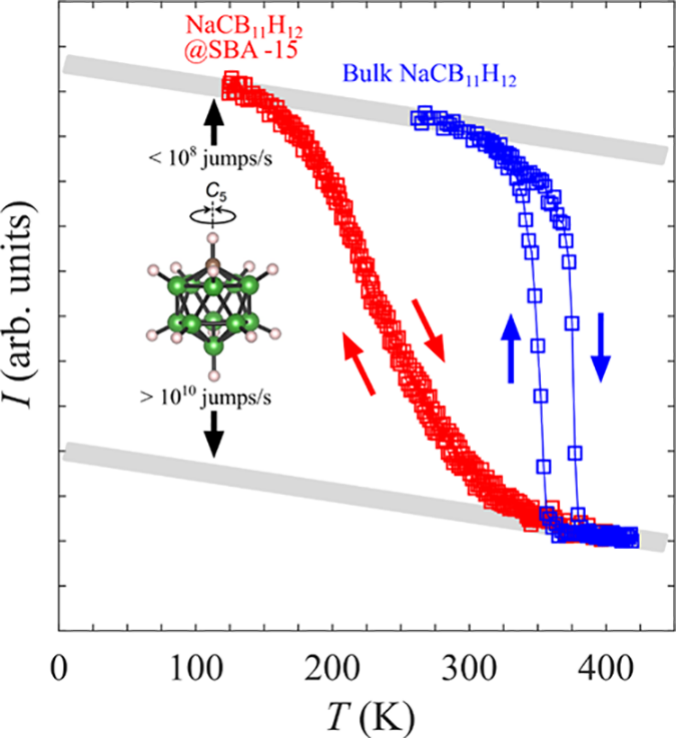
Neutron elastic-scattering FWS behavior for NaCB_11_H_12_@SBA-15 (100% loading) compared with that for bulk NaCB_11_H_12_.^10^ The gray lines indicate the decrease in the integrated FWS intensity related to the Debye–Waller factor summed over detectors covering a *Q* range of 0.87 to 1.68 Å^–1^. Arrows differentiate heating and cooling scans. For a reasonable qualitative comparison, the individual data sets were scaled so as to have similar minimum and maximum intensities. Inset: reorienting CB_11_H_12_^–^ anion with C, B, and H atoms denoted by brown, green, and white spheres, respectively.

QENS measurements were conducted on the nanocomposites to examine the CB_11_H_12_^–^ anion dynamics in further detail. In line with the NMR results, the nonhysteretic neutron-elastic-scattering FWSs for NaCB_11_H_12_@SBA-15 (100% loading) using HFBS (shown in Figure 7) indicate that anion reorientational jump rates enter the lower edge of the HFBS frequency window of 10^7^ jumps s^–1^ by ∼170 K, as signaled by the onset of a steeper drop in the elastic intensity above the more gradual Debye–Waller decay as the temperature is increased from 120 K. This steeper drop is a reflection of increasing quasielastic (Doppler-like) broadening of a portion of the elastic peak.^33^ At room temperature, this quasielastic component becomes too broad to further affect the elastic intensity, and the elastic intensity starts to reflatten out in the Debye–Waller fashion, indicating that the jump rates for the nanoconfined anions now well exceed the order of 10^10^ jumps s^–1^. Again, as hinted by the NMR results, the FWSs and underlying anion mobilities for NaCB_11_H_12_@SBA-15 are similar to those for the superionic Na_2_(CB_9_H_10_)(CB_11_H_12_) mixed-anion solid solution.^10^

It is clear from the heating and subsequent cooling scans for NaCB_11_H_12_@SBA-15 shown in Figure 7 that the anion mobilities exhibit nonhysteretic temperature dependence, which is consistent with the lack of any significant structural changes. This is in contrast to the hysteretic and sharper FWS decrease and increase observed for bulk NaCB_11_H_12_, which reflect the abrupt hysteretic order–disorder phase transformations between the lower-temperature, ordered orthorhombic structure (with anion jump rates <10^7^ jumps s^–1^) to the higher-temperature, disordered structures (with anion jump rates >10^10^ jumps s^–1^).

**Figure 8:**
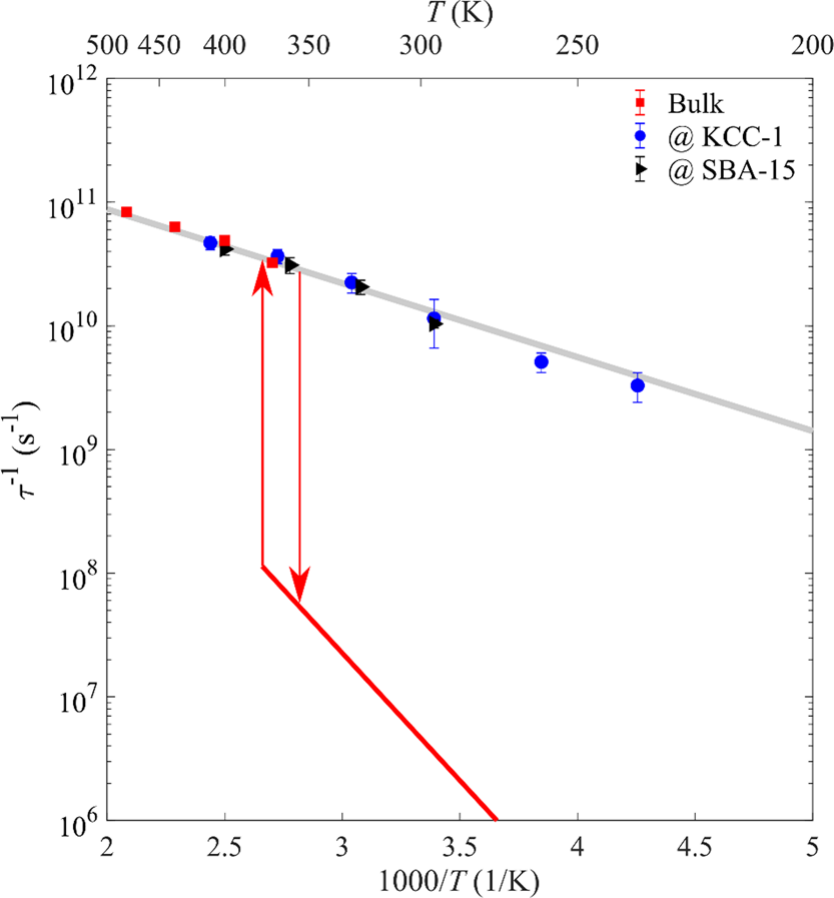
Anion reorientational jump frequencies τ_1_^–1^ for NaCB_11_H_12_ nanoconfined in SBA-15 (100% loading) and KCC-1 (150% loading) compared with those for bulk NaCB_11_H_12_,^34^ as determined by QENS. The red line shows the reorientational frequencies for the low-temperature phase of bulk NaCB_11_H_12_ determined by NMR,^31^ while the red arrows indicate the low-to-high/high-to-low phase-transition temperatures.

QENS spectra for both NaCB_11_H_12_@SBA-15 (100% loading) and NaCB_11_H_12_@KCC-1 (150% loading) typically consisted of a delta function (elastic scattering) and a Lorentzian function (quasielastic scattering), both convoluted with the instrumental resolution function above a flat baseline (Figure S6). The fundamental anion reorientational jump frequency τ_1_^–1^ inferred by the FWSs was extracted from the Lorentzian quasielastic linewidth (FWHM) Γ at low *Q* (≤0.8 Å^–1^) as a function of temperature via the relation τ_1_^–1^ = Γ/(2ℏ) and is shown in Figure 8 in comparison with that measured previously for bulk NaCB_11_H_12_.^34^ As seen, the reorientational jump frequencies of the anions in the high-temperature disordered phase of bulk NaCB_11_H_12_ (red) and both nanocomposites (blue and black) exhibit similar temperature-dependent behaviors above the bulk phase-transition temperature. This implies that SiO_2_-nanoconfined NaCB_11_H_12_ also possesses a bulk-like disordered structure in this temperature region. While the jump frequency decreases by two orders of magnitude for bulk NaCB_11_H_12_ upon cooling through the phase transition (indicated by the red arrows in Figure 8), the nanocomposite jump frequency, on the other hand, continues to extend its slower Arrhenius decrease to a low temperature, with a slope [−*E*_a_/*k*_B_; based on the relationship τ_1_^–1^ = τ_0_^–1^ exp (−*E*_a_/*k*_B_*T*), where τ_0_^–1^, *E*_a_, and *k*_B_ are the attempt frequency, activation energy, and Boltzmann constant, respectively] similar to the slope of the bulk NaCB_11_H_12_ jump frequency above its phase transition. In particular, the resulting activation energy for the combined NaCB_11_H_12_@SBA-15 and NaCB_11_H_12_@KCC-1 data over the broad temperature range of 235–410 K is 118(9) meV, which is in line with the 112 meV for bulk NaCB_11_H_12_ above the phase transition derived from previous QENS experiments.^34^

We point out that the corresponding values of the activation energies obtained from proton NMR (as detailed in the Supporting Information) are 178 meV (the average activation energy for the faster reorientational jump process in NaCB_11_H_12_@SBA-15) and 177 meV (for the high-*T* disordered phase of bulk NaCB_11_H_12_).^31^ It should be noted that the activation energies derived from QENS measurements for a number of disordered phases of polyhedral hydro(carba)borates appear to be somewhat lower than those resulting from proton NMR experiments. This feature is not uncommon, as explained in a previous study;^28^ the most probable reason for such a systematic discrepancy is the presence of a certain distribution of H jump rates.

**Figure 9:**
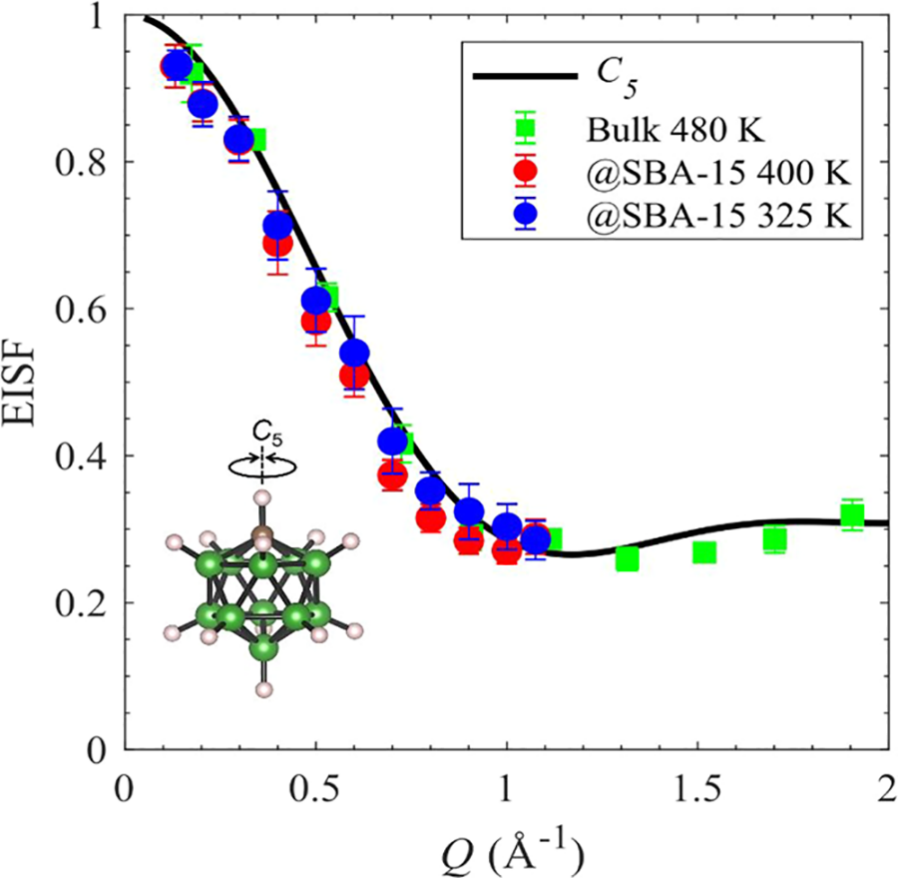
Comparison of the elastic incoherent structure factor (EISF) behavior for NaCB_11_H_12_@SBA-15 (100% loading) at 325 and 400 K with that previously reported for superionic disordered bulk NaCB_11_H_12_ at 480 K,^34^ all shown to exhibit a*Q*-dependence that matches the specific model curve (black line) expected for uniaxial fivefold (2π/5) reorientational jumps around the CB_11_H_12_^–^ anion *C*_5_ symmetry axis^35^ (see inset). Further model details can be found in the Supporting Information.

Figure 9 displays the *Q*-dependence of the elastic incoherent structure factor (EISF) for NaCB_11_H_12_@SBA-15 (100% loading) at two temperatures (400 and 325 K, which are, respectively, above and below the bulk-phase-transition temperature), compared with that reported previously for superionic disordered bulk NaCB_11_H_12_ at 480 K.^34^ The EISF is defined as the ratio of integrated neutron elastic scattering intensity to integrated total (elastic+quasielastic) scattering intensity (see Figure S6 in the Supporting Information for further details). The good agreement with the model curve shown in Figure 9 indicates that the CB_11_H_12_^–^ anions in the disordered bulk NaCB_11_H_12_ preferentially undergo uniaxial fivefold (2π/5) reorientational jumps around the CB_11_H_12_^–^ anion *C*_5_ symmetry axis. It is also apparent that the anions in the nanoconfined NaCB_11_H_12_ follow this same *C*_5_ model curve, both above and below the bulk order–disorder transition temperature. This not only further corroborates the dynamical similarities between the nanoconfined NaCB_11_H_12_ (over the entire temperature range probed) and bulk NaCB_11_H_12_ above its phase transition but also confirms that all CB_11_H_12_^–^ anions in the nanoconfined NaCB_11_H_12_ remain highly mobile, even below the bulk phase-transition temperature. Indeed, any immobile, bulk-like fraction present in the nanopores would result in an anomalous increase in the measured EISF values at 325 K compared to those at 400 K and those for bulk NaCB_11_H_12_ at 480 K (Figure 9) because of an increased fraction of the elastic scattering intensity originating from such an immobile fraction.

The combined NMR and QENS dynamical results are in line with the PXRD structural results, all pointing to the stabilization of a disordered bulk-like phase for SiO_2_-nanoconfined NaCB_11_H_12_ over the entire studied temperature range, down to cryogenic temperatures.

### Cationic Conductivity

**Figure 10:**
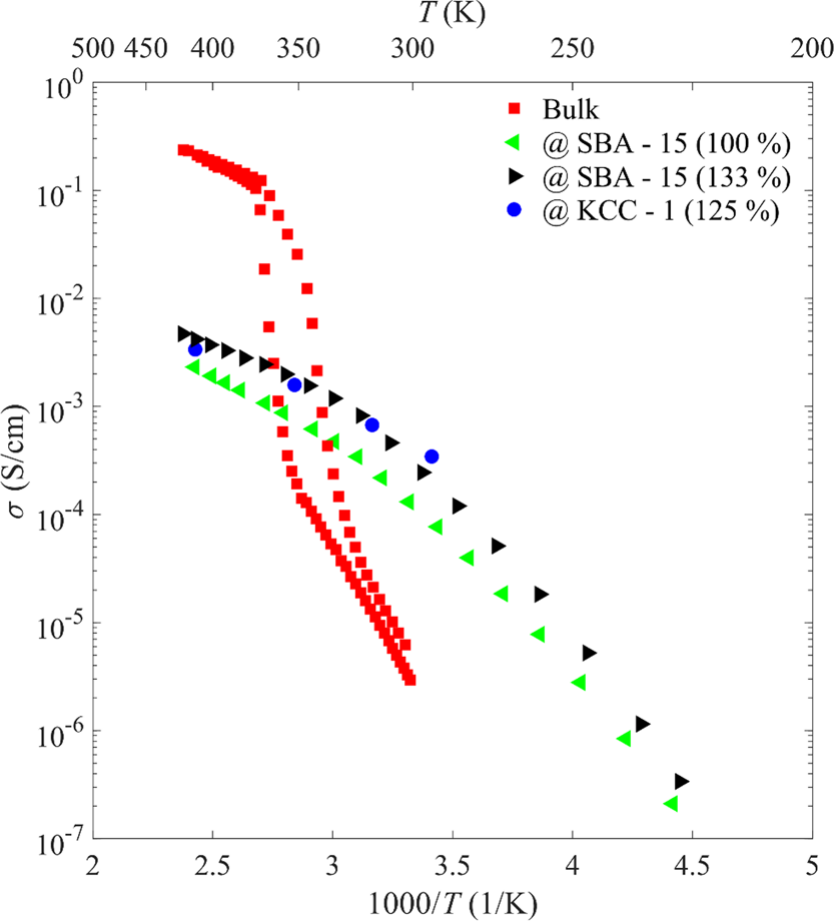
Na^+^ cationic conductivities extracted from impedance spectroscopy for NaCB_11_H_12_@SBA-15 (100 and 133% loading; green and black triangles) and NaCB_11_H_12_@KCC-1 (125% loading; blue circles) as compared with those for bulk NaCB_11_H_12_ (red squares). In contrast to the hysteresis in conductivity observed for bulk NaCB_11_H_12_, none is evident for the nanocomposites. Representative Nyquist plots are shown in the Supporting Information, along with details for extracting the conductivities.

The Na^+^ ionic conductivities for three nanocomposites, NaCB_11_H_12_@SBA-15 (100% and 133% loadings) and NaCB_11_H_12_@KCC-1 (125% loading), are compared with those for bulk NaCB_11_H_12_ (Figure 10). For the bulk (red), two regions can be observed: a high-temperature region, where the sample is in its dynamically disordered phase with superionic conductivity (∼1 × 10^–1^ S cm^–1^), and a low-temperature region, where the ionic conductivity is poor (∼1 × 10^–5^–1 × 10^–6^ S cm^–1^) because of the sample being in its ordered orthorhombic phase. Separating these two regimes is a phase-transition region where the conductivity changes drastically. The behavior of the nanoconfined NaCB_11_H_12_ (green, blue, and black) is utterly different; while the materials exhibit lower conductivities at higher temperatures (∼2 × 10^–3^–6 × 10^–3^ S cm^–1^), there is no pronounced drop at lower temperatures, and the conductivities remain relatively high at room temperature (∼1 × 10^–4^–3 × 10^–4^ S cm^–1^). This suggests that a highly conductive, dynamically disordered phase of NaCB_11_H_12_ was stabilized by the formation of the nanocomposite. Based on a linear least-squares fit of the plot of ln(σ*T*) vs *T*^–1^ shown in Figure S8 for the nanocomposite conductivity data (Figure 10) (133%-loaded SBA-15), a conductivity activation energy *E*_c_ of 216 meV was determined from the slope (−*E*_c_/*k*_B_) in the temperature range 330–420 K. Below 285 K, a second linear region can be observed with an activation energy of 567 meV. Separating these two regimes is a temperature-transition region (∼285–300 K) where the slope gradually changes from the high-temperature value to the low-temperature value. This two-regime behavior and the corresponding temperature-transition region near the ambient temperature are similar to those observed previously for Na_2_(CB_9_H_10_)(CB_11_H_12_).^10^ Like the *closo*-anions in this latter mixed-anion compound, the reorientational jump rate frequencies of the CB_11_H_12_^–^ anions in NaCB_11_H_12_@SiO_2_ in the temperature-transition region are of the order of 10^10^ jumps/s, which is only an order of magnitude or so higher than the translational jump frequencies of the Na^+^ cations.^30,31,36^ Thus, although still significantly higher than the cation translational jump frequencies in this region, it is likely that further below these temperatures, the anion reorientational jump frequencies become too low and will no longer have such a marked synergistic dynamical effect^34^ on the cation diffusion, as is the case at higher temperatures. This will ultimately lead to a change in the rate-limiting step, as reflected in the observed increase in *E*_c_ to 567 meV. It is worth pointing out that this activation energy for conductivity is significantly larger than that determined for Na^+^ diffusion by NMR (162 meV) for this corresponding temperature region. However, this is reasonable and commonplace^2,4^ because the NMR value reflects the local (microscopic) conditions, while *E*_c_ reflects the global (macroscopic) conditions that include such additional effects as grain boundaries, pore-filling factors, and nanoparticle/cluster connectivity. The discrepancy in barriers indicates that *E*_c_ could potentially be improved to approach more closely the NMR value by addressing and mitigating the abovementioned global conductivity bottlenecks.

It should be noted that, although solution loadings above 100% shown here lead to dried nanocomposites with a minor amount of bulk NaCB_11_H_12_ crystallites outside the nanopores, the threefold poorer conductivities for 100%-loaded SBA-15 compared to 133%-loaded SBA-15 shown in Figure 10 indicate that some excess bulk NaCB_11_H_12_ is desirable to improve the ultimate ionic conductivities by presumably acting as a relatively thin bridge for enhancing the connectivity between otherwise contiguous clusters of the nanoconfined NaCB_11_H_12_ material from different neighboring SiO_2_ scaffold particles. Such a strategy was successfully used previously for nanoconfined LiBH_4_ systems.^15^

In our studies, although increasing the loadings above the 100% level was found to benefit the conductivities above the bulk phase-transition temperature, above a certain point, it proved to be detrimental to the conductivities below the bulk-phase-transition temperature because of excessive amounts of poorly conductive bulk material separating neighboring SiO_2_ particles. For example, we observed that increasing the loading of NaCB_11_H_12_@KCC-1 from 125 to 150% led to a similar favorable conductivity at 410 K (above the bulk phase-transition temperature) but a sixfold lower conductivity compared to that for 125% loading at room temperature (below the bulk phase-transition temperature).

Although it appears that the highly conductive, disordered NaCB_11_H_12_ phase is stabilized within the SiO_2_ nanopores over a wide temperature range, it is also clear that various extraneous factors cause the ultimate conductivity of the pelletized nanocomposites to be more than an order of magnitude lower than that for bulk NaCB_11_H_12_ above its phase-transition temperature. In addition to the deleterious effect of incomplete conductive connectivity pathways that can occur between different infiltrated SiO_2_ particles, the mere presence of the insulating SiO_2_ scaffold material dilutes the otherwise conductive NaCB_11_H_12_, in effect, reducing the composite conductivity based on the overall cross-sectional area of the pellet. Also, because the infiltration method is solution-based instead of melt-based, the removal of the solvation water after 100% solution infiltration necessarily leaves about 40% void space in the dry-NaCB_11_H_12_-infused nanopores (based on saturated-solution and anhydrous NaCB_11_H_12_ densities), which likely causes further significant degradation in conductivity. Finally, the resulting pellet density after the compression of both SBA-15- and KCC-1-based samples is found to be roughly two times less than expected based on the densities of SiO_2_ and NaCB_11_H_12_, which means that there is a substantial amount of extra void space accumulated between the SiO_2_ scaffold particles, in addition to that formed within the nanopores from water removal. For example, assuming that the remaining nanopore void space stays intact after pellet compression, the 100%-loaded SBA-15 should have a theoretical pellet density of ∼1.20 g cm^–3^ (and 1.76 g cm^–3^, assuming that the nanopore voids collapse; see details in the Supporting Information), whereas its actual density was 0.71 g cm^–3^. This suggests that about 40% void space exists in the 100%-loaded SBA-15 sample. Assuming that the extra bulk NaCB_11_H_12_ in 133%-loaded SBA-15 fills some of these voids, the density is expected to increase to 0.80 g cm^–3^, which compares well with the actual density of 0.79 g cm^–3^ for the 133%-loaded SBA-15 sample.

Strategies that can increase NaCB_11_H_12_ connectivity and reduce the fraction of void space should lead to significant improvements in nanocomposite conductivities. These might include using nanoporous silica with different morphologies, larger pore volume fractions, and more favorable compaction properties, as well as performing multiple infiltration/drying treatments with saturated salt solution to minimize intrapore voids.

It is worth noting that the observed nanoconfinement behavior for NaCB_11_H_12_ is somewhat different from that found for LiBH_4_. In contrast to NaCB_11_H_12_, multiple studies of LiBH_4_ nanoconfined in carbon, silica, and alumina scaffolds^14,15,37−43^ down to subambient temperatures indicate that only a fraction of the infiltrated material near the nanopore walls behaves in a nonbulk-like fashion, with highly reorientationally mobile BH_4_^–^ anions and highly diffusive Li^+^ cations, while the remaining fraction near the nanopore center displays more bulk-like behavior with much lower anion and cation mobilities. Moreover, unlike nanoconfined NaCB_11_H_12_, where no translational mobility of the otherwise rotationally liquid CB_11_H_12_^–^ anions is observed by NMR, long-range diffusive motion of the BH_4_^–^ anions is indeed observed for the fraction of nanoconfined, nonbulk-like LiBH_4_ residing near the nanopore wall,^38^ suggesting the presence of a relatively more fluidic LiBH_4_ interface layer and a possibly different conduction mechanism. It is thought that this highly conductive LiBH_4_ layer results from compound-altering interactions with the nanopore wall and may involve the formation of an interfacial space-charge region.^44−46^

In addition to the differences with nanoconfined LiBH_4_, the intriguing results shown here for NaCB_11_H_12_@SiO_2_ are also qualitatively dissimilar to the previously mentioned behavior for SiO_2_-nanoconfined Li_2_B_12_H_12_,^17,18^ where most, if not all, Li_2_B_12_H_12_ was in a bulk-like ordered α-phase at ambient temperatures, with only a very minor fraction possibly in a highly conductive disordered β-phase normally only stable at much higher temperatures. Based on the known pore-size-dependent bifurcated phase behavior for nanoconfined LiBH_4_ systems,^40,41^ it is reasonable to assume that Li_2_B_12_H_12_ might also form a thin disordered-phase layer at the Li_2_B_12_H_12_-SiO_2_ interface with a predominant core of bulk-like Li_2_B_12_H_12_, even if such bifurcation does not appear to be the case for NaCB_11_H_12_@SiO_2_ for similar nanopore sizes. Nonetheless, whether or not the apparently unremarkable nanoconfined-Li_2_B_12_H_12_ behavior is specific to particular (carba)borates and/or cations is a subject for further studies. Moreover, although the disordered NaCB_11_H_12_ polymorphs are superionic phases and are therefore clearly a major contributor to the favorable NaCB_11_H_12_@SiO_2_ nanocomposite conductivities found in this study, at this time, we still do not know to what extent NaCB_11_H_12_@SiO_2_ interface effects enable enhanced Na^+^ translational mobilities that may also be contributing.

## Conclusions

Using a combination of techniques, we have shown that by depositing NaCB_11_H_12_ into nanoporous, high-surface-area silica via a simple aqueous salt infiltration method, it is possible to stabilize a bulk-like superionic phase down to cryogenic temperatures. This contrasts with bulk NaCB_11_H_12_, which can maintain its disordered superionic polymorphs only above ∼354 K. Even without any further optimization strategies, the nanocomposites presented here show a significantly higher cationic conductivity at room temperature (3 × 10^–4^ S cm^–1^) than for bulk NaCB_11_H_12_ (3 × 10^–6^ S cm^–1^) as well as for the best nanoconfined-LiBH_4_-based composites in SiO_2_ and Al_2_O_3_ (∼1 × 10^–4^ S cm^–1^).^14−16^ The conductivity of NaCB_11_H_12_@SiO_2_ can likely be further improved by a variety of factors, such as multiple solution loading/drying cycles and/or optimizing the choice of nanostructure used to stabilize the superionic phase. This could potentially solve two of the current issues: insufficient filling of salt in the nanocomposite, leading to less than full conductive connectivity, and the formation of excessive bulk NaCB_11_H_12_ coating the nanostructures and thus acting as a resistive layer at lower temperatures. We believe that fused nanoporous SiO_2_ macrostructures formed via sol–gel or similar synthesis processes to yield interconnecting porous channels, with no external nanoparticle surfaces, could ultimately prove to be better scaffold candidates. Finally, because ball-milling of NaCB_11_H_12_ and other polyhedral (carba)borate salts has been previously shown to promote the formation of high-temperature superionic disordered phases at ambient temperatures, a process that was further enhanced by ball-milling with other compounds,^9^ it may yet turn out to be worthwhile to ball-mill these types of salts together with either nanoporous or high-surface-area nonporous SiO_2_ or Al_2_O_3_, as has already been done successfully with LiBH_4_,^15,45,46^ instead of the current solution infiltration procedure. If intermixed finely enough and in the right proportions, the resulting compressed composites could have fully connective three-dimensional networks of superionic salt nanoconfined and stabilized among the oxide scaffold particles, with reduced void volume and thus improved overall conductivity.

## Supporting Information Available

Complementary DSC, PXRD, NVS, NMR, QENS, and impedance data (PDF)
