# Supplementary material for: Promoting Persistent Superionic Conductivity in Sodium Monocarba-closo-dodecaborate NaCB11H12 via Confinement within Nanoporous Silica
Source: J Phys Chem C Nanomater Interfaces. 2021 Jul 26;125(30):16689–99. doi: 10.1021/acs.jpcc.1c03589 (PMC8392346; doi:10.1021/acs.jpcc.1c03589)
Supplement: Supplementary file 1 — jp1c03589_si_001.pdf [file jp1c03589_si_001.pdf]

Supporting Information

Promoting Persistent Superionic Conductivity in Sodium Monocarba-*closo*-dodecaborate $NaCB_{11}H_{12}$ via Confinement within Nanoporous Silicas

Mikael S. Andersson,[*,∝,∇,†] Vitalie Stavila,[§] Alexander V. Skripov,[*,Δ] Mirjana Dimitrievska,[†,⊥,#] Malgorzata T. Psurek,[†,◊] Juscelino B. Leão,[†] Olga A. Babanova,[Δ] Roman V. Skoryunov,[Δ] Alexei V. Soloninin,[Δ] Maths Karlsson,[∝] and Terrence J. Udovic[*,†,‡]

[∝]Department of Chemistry and Chemical Engineering, Chalmers University of Technology, SE-412 96 Göteborg, Sweden

[∇]Department of Chemistry, Ångström Laboratory, Uppsala University, Box 538, 75121 Uppsala, Sweden

[†]NIST Center for Neutron Research, National Institute of Standards and Technology, Gaithersburg, MD 20899-6102, United States

[§]Energy Nanomaterials, Sandia National Laboratories, Livermore, CA 94551, United States

[Δ]Institute of Metal Physics, Ural Branch of the Russian Academy of Sciences, Ekaterinburg, 620108, Russia

[⊥]National Renewable Energy Laboratory, Golden, CO 80401, United States

[#]Laboratory of Semiconductor Materials, Institute of Materials, Ecole Polytechnique Fédérale de Lausanne, 1015 Lausanne, Switzerland

[◊]Department of Chemistry, University of Maryland, College Park, MD 20742, United States

[‡]Department of Materials Science and Engineering, University of Maryland, College Park, MD 20742, United States

*Authors to whom correspondence should be addressed. E-mail: mikael.andersson@kemi.uu.se; skripov@imp.uran.ru; udovic@nist.gov

DSC

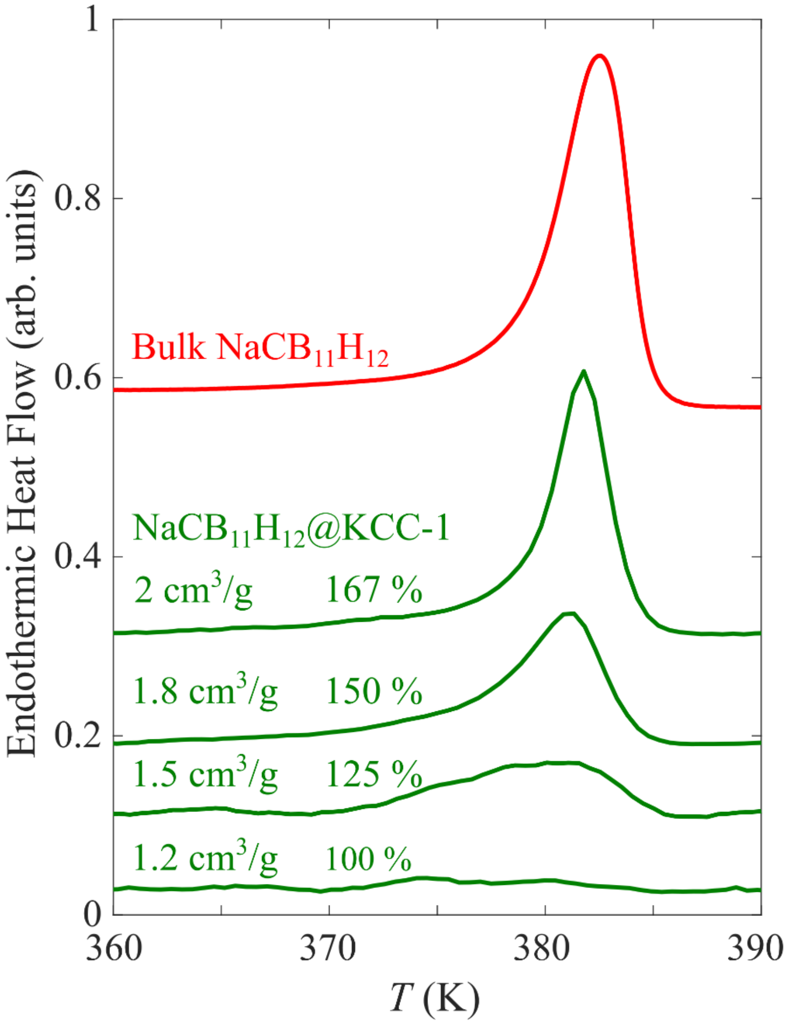

Figure S1. Exemplary DSC scans in heating (5 K min^{-1}) of dried $NaCB_{11}H_{12}$@KCC-1 nanocomposite samples derived from different saturated $NaCB_{11}H_{12}$ solution loadings (cm^3/g and corresponding percentage of available pore volume filled) of KCC-1, compared with that for bulk $NaCB_{11}H_{12}$. All nanocomposite scans are normalized with respect to the same mass of KCC-1.

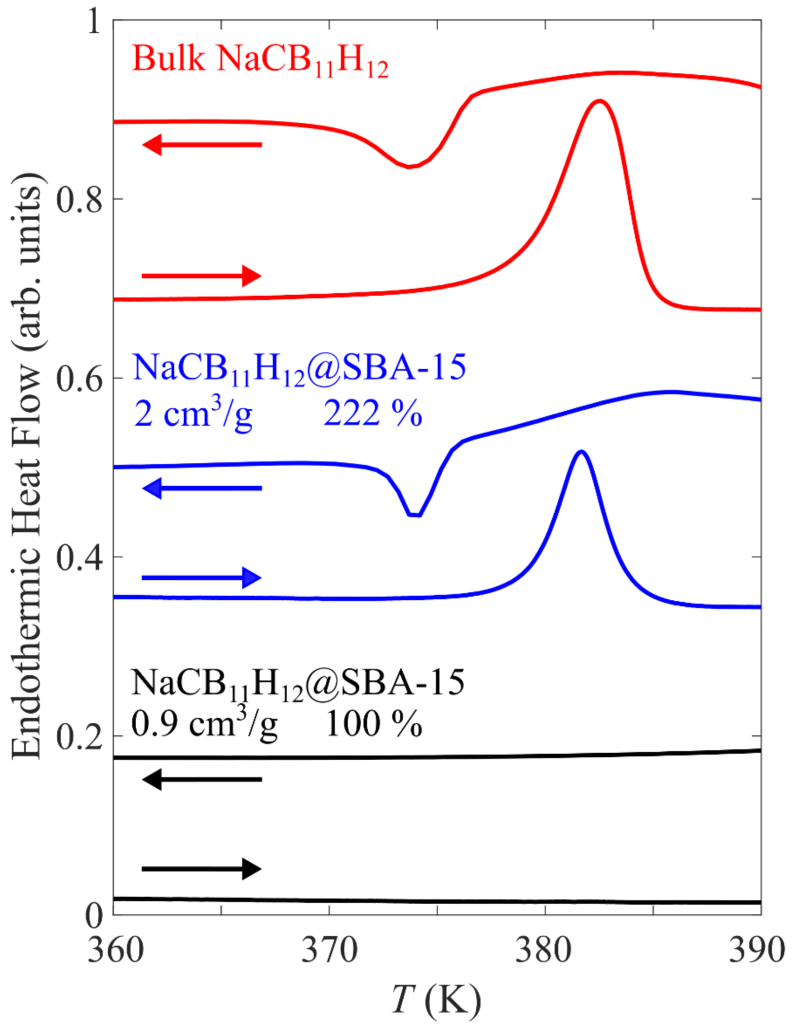

Figure S2. Exemplary DSC scans in heating (5 K min^{-1}) and in cooling (1.4 K min^{-1}) of dried $NaCB_{11}H_{12}$@SBA-15 nanocomposite samples derived from different saturated $NaCB_{11}H_{12}$ solution loadings (cm^3/g and corresponding percentage of available pore volume filled) of SBA-15, compared with that for bulk $NaCB_{11}H_{12}$. All nanocomposite scans are normalized with respect to the same mass of SBA-15.

PXRD

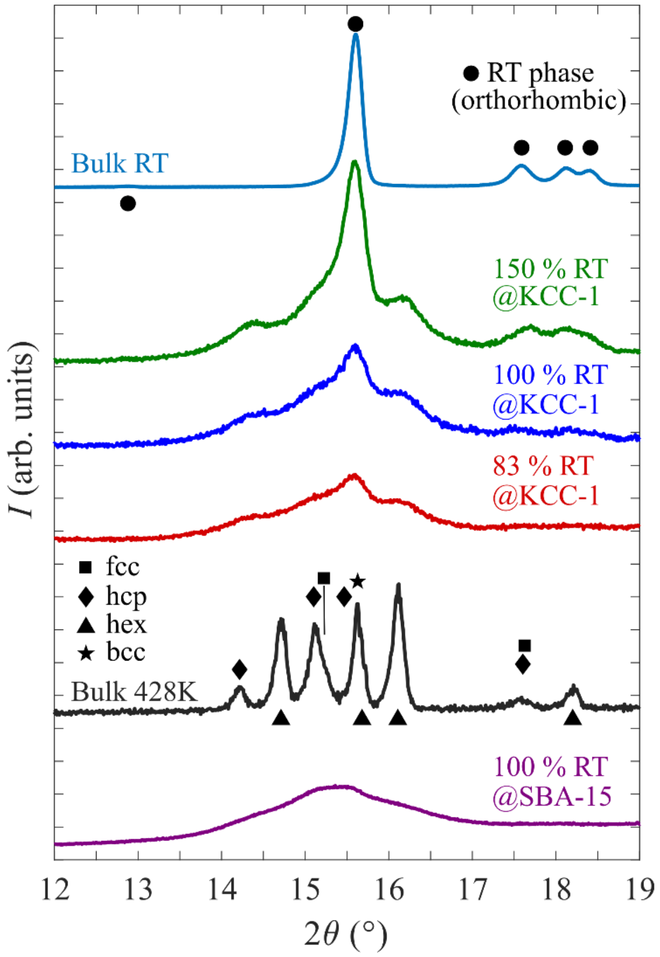

Figure S3. PXRD patterns for dried $NaCB_{11}H_{12}$@KCC-1 nanocomposites for different solution loadings at room temperature compared with that for 100 % $NaCB_{11}H_{12}$@SBA-15 as well as those for bulk $NaCB_{11}H_{12}$ from Ref. S1.

Compared to the 100 % $NaCB_{11}H_{12}$@SBA-15 pattern, the 100 % and 83 % $NaCB_{11}H_{12}$@KCC-1 patterns are consistent with a somewhat higher selectivity for the hexagonal, hcp, and bcc polymorphs (at the expense of the fcc polymorph). Since the bcc polymorph peak position is close to that for the room-temperature bulk position, we cannot rule out some lingering amount of bulk phase for 100 % and 83 % $NaCB_{11}H_{12}$@KCC-1 due to less than fully homogenized mixing of solution and KCC-1 during synthesis, although the DSC scans show no obvious bulk-like transition peak. Even though the different polymorphs are assumed to be close in energy, their relative stabilities are known to be dependent on temperature[S1] and one may reasonably speculate that they may also be dependent on crystallite size. For $NaCB_{11}H_{12}$@SiO_2, the increased preference for the other polymorphs in KCC-1 may thus be related to the formation of larger $NaCB_{11}H_{12}$ nanocrystallites, on average, in KCC-1 than in SBA-15, since KCC-1 possesses a relatively larger average pore size and a broader pore size distribution than SBA-15.[S2]

NVS

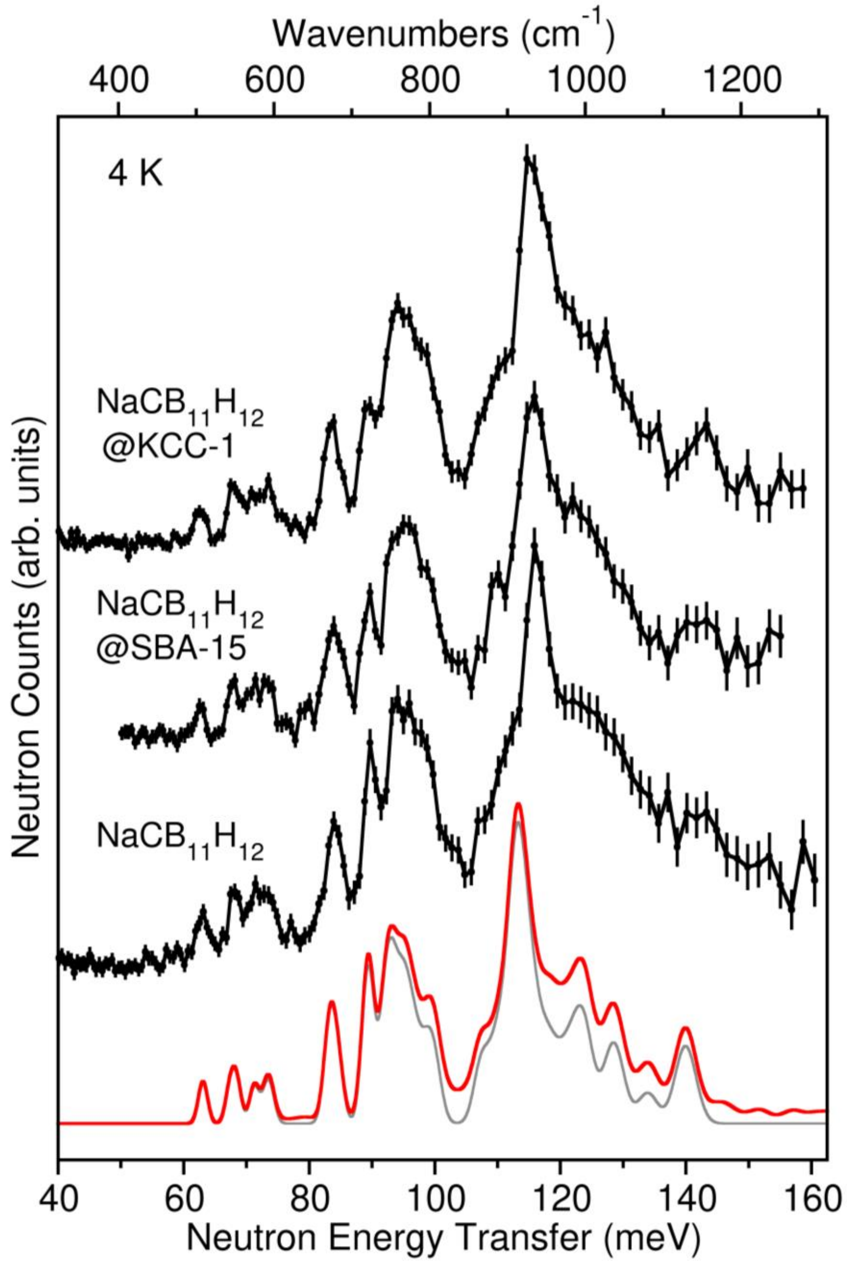

Figure S4. Neutron vibrational spectra at 4 K for $NaCB_{11}H_{12}$@SBA-15 and $NaCB_{11}H_{12}$@KCC-1 (100 % loadings) compared with that from Ref. S1 for bulk $NaCB_{11}H_{12}$. Below (also from Ref. S1) are the simulated one-phonon (gray) and one+two-phonon densities of states (red) from first-principles phonon calculations of the optimized orthorhombic structure. (N.B., 1 meV = 8.066 cm^{-1}.)

NMR

For parametrization of the proton spin-lattice relaxation rate data presented in Figure 4, we have to choose an appropriate model. According to the standard model for motionally-induced spin-lattice relaxation,[S3] in the limit of slow motion ($\omega\tau \gg 1$), R_1^H should be proportional to $\omega^{-2}\tau^{-1}$, and in the limit of fast motion ($\omega\tau \ll 1$), it should be proportional to τ, being frequency-independent. If the hydrogen jump rate τ^{-1} follows a regular Arrhenius-type temperature dependence with the activation energy E_a,

$$\tau^{-1} = \tau_0^{-1} \exp(-E_a/k_B T), \qquad \text{(eqn. S1)}$$

a plot of ln R_1^H vs. T^{-1} is expected to be linear in the limits of both slow and fast motion with the slopes of $-E_a/k_B$ and E_a/k_B, respectively. As can be seen from Figure 4, the experimental R_1^H (T) data exhibit strong deviations from the predictions of the standard model. In particular, the low-temperature slope of the R_1^H (T) peak is found to change significantly near 140 K. Furthermore, the frequency dependence of R_1^H at low temperatures is considerably weaker than the predicted ω^{-2} dependence for the limit of slow motion. These features suggest a coexistence of at least two jump processes, each of which is characterized by different motional parameters and a certain distribution of the jump rates.[S4] It should be noted that two types of reorientations characterized by different jump rates were previously found to coexist in a number of borohydrides[S5,S6,S7] and *closo*-hydroborates.[S8]

The simplest model for parametrization of the R_1^H (T) data in such a case is the two-peak model with a Gaussian distribution of the activation energies.[S5] The parameters of the model are the average activation energies $\overline{E}_{ai}$, the widths (dispersions) ΔE_{ai} of the activation energy distributions, the pre-exponential factors τ_{0i}, and the amplitude parameters ΔM_i reflecting the strength of fluctuating parts of dipole-dipole interactions of ^{1}H spins for each of the motional processes i (i = 1, 2). These model parameters have been varied to find the best fit to the R_1^H (T) data at two resonance frequencies *simultaneously*. The results of this simultaneous fit to the experimental data in the range 80 K to 426 K are shown by solid lines in Figure 4; the corresponding motional parameters are $\overline{E}_{a1}$ = 178(9) meV, ΔE_{a1} = 45(2) meV, τ_{01} = 2.3(1) × 10^{-14} s, ΔM_1 = 1.5(1)×10^9 s^{-2} for the faster process, and $\overline{E}_{a2}$ = 206(10) meV, ΔE_{a2} = 22(1) meV, τ_{02} = 1.0(1) × 10^{-13} s, ΔM_2 = 4.0(2)×10^9 s^{-2} for the slower process. Note that the average value of the activation energy for the faster process in $NaCB_{11}H_{12}$/SBA-15 is very close to the activation energy for reorientations in the high-T disordered phase of bulk $NaCB_{11}H_{12}$ (177 meV).[S9]

The amplitude of the R_1^{Na} (T) peak shown in Figure 5 is too high to be ascribed to dipole-dipole interactions between nuclear spins. This leads us to the conclusion that the observed ^{23}Na spin-lattice relaxation rate is dominated by the quadrupole mechanism related to electric-field-gradient (EFG) fluctuations at ^{23}Na sites. However, anion reorientations cannot be responsible for the dominant quadrupole relaxation mechanism, since each reorientational jump does not change the charge configuration around ^{23}Na sites. On the other hand, any jump of Na^+ from one site to another is expected to change the principal EFG value and/or the angle between the

principal axis of the EFG tensor and the magnetic field direction, providing the effective quadrupole relaxation mechanism. Thus, the R_1^{Na} (T) peak near 270 K indicates that the diffusive Na^+ jump rate τ_d^{-1} reaches approximately $\omega \sim 8 \times 10^8$ s^{-1} at this temperature. Such a result is consistent with the high room-temperature ionic conductivity observed for $NaCB_{11}H_{12}$/SBA-15. We have tried to parametrize only the R_1^{Na} (T) data below 270 K, since the relaxation rate peak at higher temperatures is only partially revealed (see Figure 5). The simplest approach to describe the motionally induced quadrupole contribution to the spin-lattice relaxation rate is based on the expression

$$R_1^{\mathrm{Na}} = \frac{M_Q}{\omega}\left[\frac{y}{1+y^2} + \frac{4y}{1+4y^2}\right], \qquad \text{(eqn. S2)}$$

where $y = \omega\tau_d$, and the Arrhenius law for τ_d with the activation energy E_a^d for Na^+ diffusive motion. Here the amplitude factor M_Q is proportional to the square of the electric quadrupole moment of ^{23}Na and the mean square of the fluctuating part of EFG at ^{23}Na sites due to diffusive jumps. The solid line in Figure 5 shows the fit of this model to the experimental R_1^{Na} (T) data over the range of 150 – 270 K; the corresponding parameters are E_a^d = 162(4) meV, τ_{d0} = 7.8(2) $\times 10^{-13}$ s, and M_Q = 3.3(1) $\times 10^{11}$ s^{-2}. It can be noted that E_a^d is significantly smaller than the corresponding activation energy for the Na^+ ionic conductivity (E_c = 567 meV) for the corresponding temperature interval (see Fig. S8).

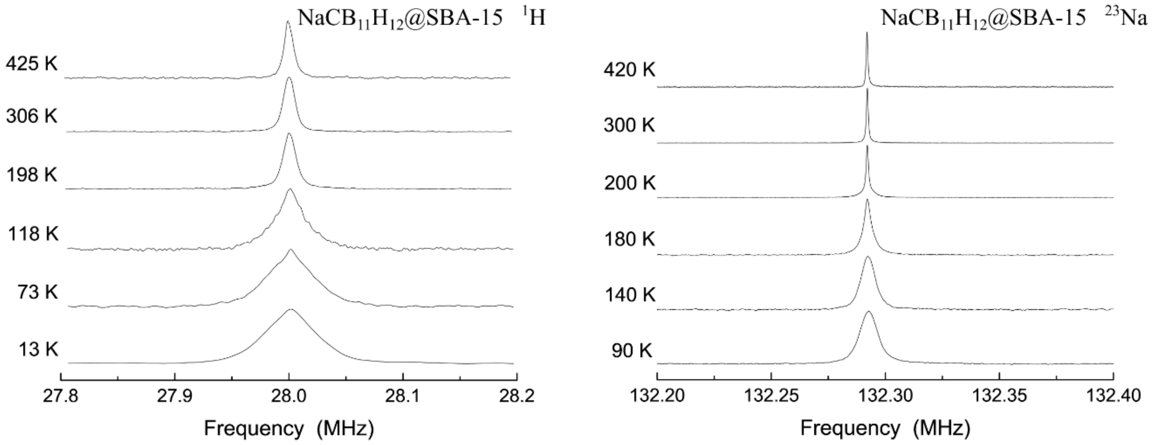

Figure S5. Evolution of the measured ^1H and ^{23}Na NMR spectra with temperature for $NaCB_{11}H_{12}$@SBA-15 (100 % loading).

QENS fits

To extract the fundamental reorientational jump frequency, τ_1^{-1}, the QENS spectra were fitted to the scattering function $S(Q,\omega) = R(Q,\omega) \otimes [\delta(Q,\omega) A_E(Q) + \sum L_i(Q,) A_{QE,i}(Q)]$, where Q is the momentum transfer of the neutron, $E = \hbar\omega$ is the energy transfer of the neutron, ω is the angular frequency, $\hbar$ is the reduced Planck constant, R is the instrumental resolution function, δ is a delta function, L_i is a Lorentzian function corresponding to index i, and A_E and A_{QE} are the integrated elastic and quasielastic scattering intensities corresponding to the respective delta and Lorentzian functions. The Q intervals used for the fits were Q_{min} *to* Q = 0.8 Å^{-1}. Q_{min} = 0.069 Å^{-1} for λ = 8 Å and Q_{min} = 0.055 Å^{-1} for λ = 10 Å. An example of a fit is shown in Figure S6 for $NaCB_{11}H_{12}$@SBA-15 at 360 K.

EISF for 5-fold reorientations

The elastic incoherent structure factor for 5-fold reorientations of the $CB_{11}H_{12}^-$ anion can be calculated using the relation $\mathrm{EISF}_{C_5} = \frac{1}{6} + \frac{1}{6}[1 + 2j_0(Qd_1) + 2j_0(Qd_2)]$, where j_0 is the zeroth order spherical Bessel function, and d_1 and d_2 are the two jump lengths involved in a 5-fold reorientation (relative to the starting position).[S10]

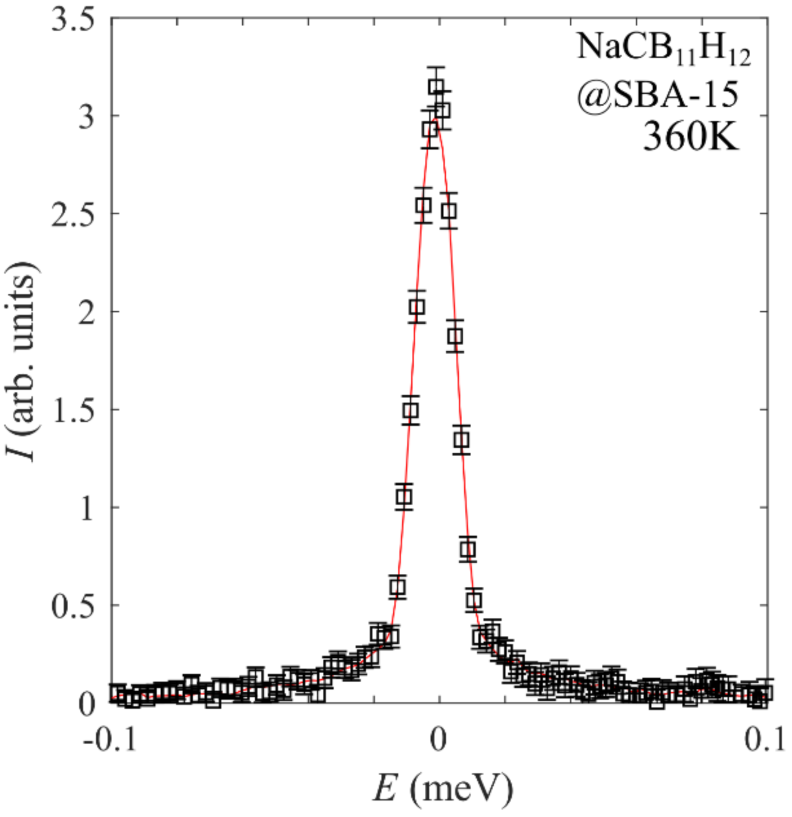

Figure S6. QENS spectra for $NaCB_{11}H_{12}$@SBA-15 (100 % loading) at 360 K with corresponding fit. Error bars are one standard deviation.

Theoretical pellet densities

The theoretical pellet densities of the pellets used for the impedance measurements were calculated using the method described below. Theoretical compressed pellet densities are defined as the highest attainable density assuming that the SiO_2 particles "fuse" together to remove all extra-particle void volume while keeping the nanoporous structure intact. These densities are calculated based on a saturated $NaCB_{11}H_{12}$+5.5 H_2O solution density of 1.11 g/cm^3, a bulk orthorhombic $NaCB_{11}H_{12}$ phase density of 1.182 g/cm^3,[S1] a disordered $NaCB_{11}H_{12}$ phase density of 1.101 g/cm^3,[S1] and an assumed SiO_2 density of 2.20 g/cc (the density of amorphous SiO_2).[S11]

As an example, for 100 %-loaded SBA-15, if we start with a basis of 1 g of SBA-15 with 0.9 cm^3/g of saturated solution fully loading the nanopores, then:

(1g SiO_2)/(2.20 g/cm^3) = 0.455 cm^3 SiO_2

(0.9 cm^3 sat. sol.)(1.11 g/cm^3) = 0.999 g sat. solution

mol wt. $NaCB_{11}H_{12}$ = 166.01 g/mol

mol wt. $NaCB_{11}H_{12}$+5.5H_2O = 265.09 g/mol

mass fraction of $NaCB_{11}H_{12}$ in sat. solution = 166.01/265.09 = 0.626 g $NaCB_{11}H_{12}$/g sat. sol.

Total nanocomposite mass = 1 g SiO_2 + (0.999 g sat. sol.)(0.626) = 1 g SiO_2 + 0.625 g $NaCB_{11}H_{12}$ = 1.625 g

Total volume = 0.455 cm^3 SiO_2 + 0.9 cm^3 nanopore volume = 1.355 cm^3

Therefore, theoretical density (100%-loaded SBA-15) = 1.625 g/1.355 cm^3 = 1.20 g/cm^3.

Impedance spectroscopy

Figure S7 shows the AC impedance spectra for a few select temperatures for $NaCB_{11}H_{12}$@SBA-15 (133 % loading). The resistances of the sample were estimated by fitting the high-frequency semicircle using an equivalent circuit of a resistance and a capacitance in parallel. At higher temperatures where no semicircle could be observed, the resistance was estimated from the intercept of the high-frequency tail.

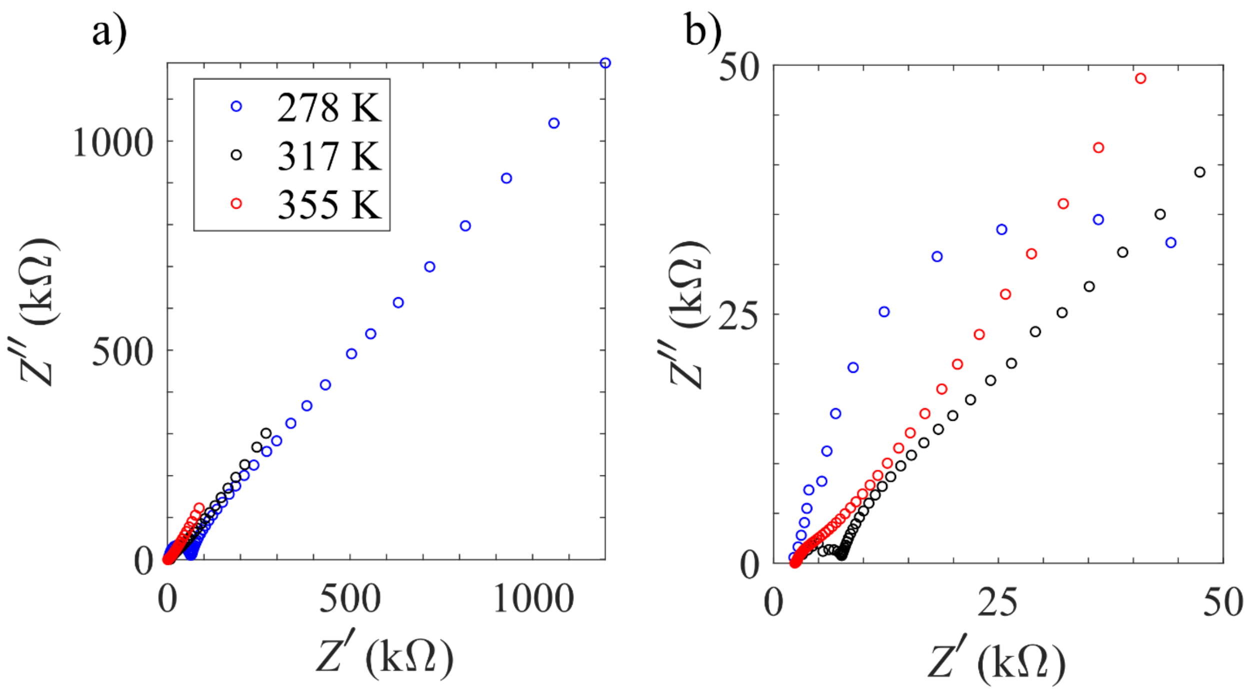

Figure S7. a) AC impedance spectra for $NaCB_{11}H_{12}$@SBA-15 (133 % loading) at 278 K, 317 K and 355 K. b) An enlarged section of the data presented in a).

Activation energy of the cation conductivity

Figure S8 shows a plot of ln(σ*T*) vs 1000/*T* for $NaCB_{11}H_{12}$@SBA-15 (133 % loading) from which the activation energy E_a of the cation conductivity has been extracted from the slope ($-E_a/k_B$).

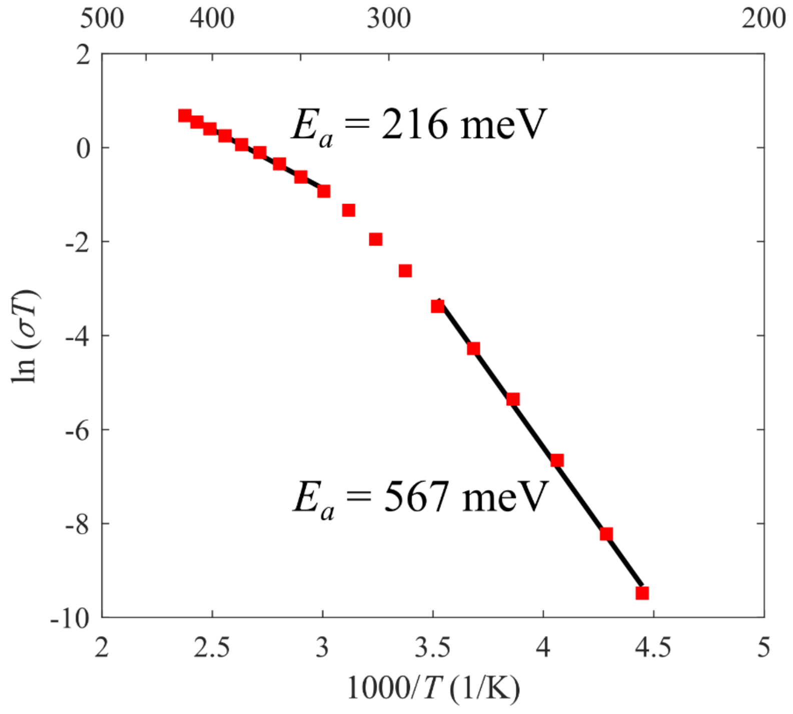

Figure S8. ln(σ*T*) vs 1000/*T* for $NaCB_{11}H_{12}$@SBA-15 (133 % loading). The black lines are linear regressions of the two corresponding regions.

References

(S1) Tang, W. S.; Unemoto, A.; Zhou, W.; Stavila, V.; Matsuo, M.; Wu, H.; Orimo, S.; Udovic, T. J. Unparalleled Lithium and Sodium Superionic Conduction in Solid Electrolytes with Large Monovalent Cage-like Anions. *Energy Environ. Sci.* **2015**, *8*, 3637–3645.

(S2) Bayal, N.; Singh, B.; Singh, R.; Polshettiwar, V. Size and Fiber Density Controlled Synthesis of Fibrous Nanosilica Spheres (KCC-1). *Sci Rep.* **2016**, *6*, 24888.

(S3) Abragam, A. *The Principles of Nuclear Magnetism*, Clarendon Press: Oxford, 1961.

(S4) Markert, J. T.; Cotts, E. J.; Cotts, R. M. Hydrogen Diffusion in the Metallic Glass *a*-$Zr_3RhH_{3.5}$. *Phys. Rev. B* **1988**, *37*, 6446-6452.

(S5) Skripov, A. V.; Soloninin, A. V.; Babanova, O. A.; Hagemann, H.; Filinchuk, Y. Nuclear Magnetic Resonance Study of Reorientational Motion in α-$Mg(BH_4)_2$. *J. Phys. Chem. C* **2010**, *114*, 12370-12374.

(S6) Verdal, N.; Udovic, T. J.; Rush, J. J. The Nature of BH_4^- Reorientations in Hexagonal $LiBH_4$. *J. Phys. Chem. C* **2012**, *116*, 1614-1618.

(S7) Babanova, O. A.; Skoryunov, R. V.; Soloninin, A. V.; Dovgaliuk, I.; Skripov, A. V.; Filinchuk, Y. Nuclear Magnetic Resonance Study of Hydrogen Dynamics in $Al(BH_4)_4$-Based Hypersalts $M[Al(BH_4)_4]$ (M = Na, K, Rb, Cs). *J. Alloys Compd.* **2018**, *745*, 179-186.

(S8) Soloninin, A. V.; Dimitrievska, M; Skoryunov, R. V.; Babanova, O. A.; Skripov, A. V.; Tang, W. S.; Stavila, V.; Orimo, S.; Udovic, T. J. Comparison of Anion Reorientational Dynamics in MCB_9H_{10} and $MB_{10}H_{10}$ (M = Li, Na) via Nuclear Magnetic Resonance and Quasielastic Neutron Scattering Studies. *J. Phys. Chem. C* **2017**, *121*, 1000-1012.

(S9) Skripov, A. V.; Skoryunov, R. V.; Soloninin, A. V.; Babanova, O. A.; Tang, W. S.; Stavila, V.; Udovic, T. J. Anion Reorientations and Cation Diffusion in $LiCB_{11}H_{12}$ and $NaCB_{11}H_{12}$: ^{1}H, ^{7}Li, and ^{23}Na NMR Studies. *J. Phys. Chem. C* **2015**, *119*, 26912-26918.

(S10) Verdal, N.; Udovic, T. J.; Rush, J. J.; Cappelletti, R.; Zhou, W. Reorientational Dynamics of the Dodecahydro-*Closo*-Dodecaborate Anion in $Cs_2B_{12}H_{12}$. *J. Phys. Chem. A* **2011**,*115*, 2933–2938.

(S11) Haynes, William M., ed. (2011). *CRC Handbook of Chemistry and Physics* (92nd ed.). Boca Raton, FL: CRC Press. ISBN 1439855110.
